# Construction of Kochen–Specker Sets from Mutually Unbiased Bases

**DOI:** 10.1007/s10773-026-06472-5

**Published:** 2026-11-12

**Authors:** Mirko Navara, Karl Svozil

**Affiliations:** 1 Faculty of Electrical Engineering, Czech Technical University in Prague, Technická 2, CZ-166 27 Prague 6, Czech Republic; 2 Institute for Theoretical Physics, TU Wien, Wiedner Hauptstraße 8-10/136, 1040 Vienna, Austria

**Keywords:** Quantum contextuality, Kochen–Specker theorem, Mutually unbiased bases, Quantum logic, Orthogonality hypergraphs, Quantum foundations

## Abstract

We give a constructive analysis of Kochen–Specker (KS) contextuality at the level of complete orthogonal bases (contexts). In dimension three, three noncomputational mutually unbiased bases generate a union of 165 rays and 130 contexts. Exact provenance accounting gives a symmetric decomposition: the three 69-ray lineages share 21 rays and 10 contexts, while each contributes 48 exclusive rays and 40 lineage-specific contexts. Each 69-ray, 50-context lineage is a KS configuration, and this framework clarifies the relation among the Yu–Oh, Harding–Salinas Schmeis, and Cabello constructions. In dimensions four and five, we give parametrized orthogonality gadgets whose connector contexts force a central ray to be maximally unbiased relative to the computational basis. For the concrete four-dimensional example, the 20-ray gadget and the Cabello 18-ray set are informationally equivalent subsets of the 24-ray Peres–Mermin eigensystem, although their context hypergraphs have different two-valued-state behavior. The results show why both the rays and the complete incidence structure of their contexts are needed in assessments of KS contextuality.

## Introduction

The Kochen–Specker (KS) theorem shows that certain finite families of quantum propositions do not admit a context-independent assignment of classical truth values compatible with their orthogonality relations [1]. A finite KS proof is therefore specified not only by a set of rays but also by the complete bases in which those rays occur.

### Definition 1

(**Rays and contexts**) A *ray* is a one-dimensional subspace $[v]=\{\lambda v:\lambda \in \mathbb {C}\}$ generated by a nonzero representative $v\in \mathbb {C}^D\setminus \{0\}$. The scalar zero is included because the ray is a linear subspace. A *context* (block) in $\mathbb {C}^D$ is a set of *D* mutually orthogonal rays spanning the space. In the associated *D*-uniform hypergraph the rays are vertices and the contexts are hyperedges. A ray is *intertwining* if it belongs to at least two contexts; a ray occurring in exactly one context is nonintertwining.

### Definition 2

(**Two-valued states**) For a context hypergraph (*V*, *E*), a two-valued state is a map $s:V\rightarrow \{0,1\}$ satisfying $\sum _{v\in S}s(v)=1$ for every S∈E. A coordinatized hypergraph with no two-valued state is a KS configuration. A family of two-valued states is *separating* if every pair of distinct rays receives different values in at least one state; it is *unital* if every ray receives the value 1 in at least one state.

These definitions distinguish the logical KS criterion from resource measures used in inequalities, chromatic formulations, or nonlocal games [2–4]. Reduced incidence structures can be useful in those settings, but deleting singly occurring rays or omitting available contexts can change the set of two-valued states. For example, the seven-ray subconfiguration discussed in Ref. [5, Fig. 6(a)] has different classical-state behavior from its thirteen-ray completion. We therefore state explicitly which context hypergraph is being analyzed at each step.

The paper has two main contributions:

1. In $\mathbb {C}^3$, three noncomputational mutually unbiased bases (MUBs), together with a fixed family of algebraic generation maps, produce 225 labelled vectors. Projective identification reduces these to 165 rays, and an exhaustive orthogonality search gives 130 contexts. The provenance decomposition is symmetric: 21 rays and 10 contexts are common to all three 69-ray lineages, while each lineage has 48 exclusive rays and 40 lineage-specific contexts. Each lineage contains 69 rays and 50 contexts and is uncolorable.
2. In D=4 and D=5, connector contexts convert orthogonality into linear equations for $|x_j|^2$, forcing a central vector $u=(x_1,\ldots ,x_D)$ to be maximally unbiased relative to the computational basis. In the concrete D=4 case, the resulting 20-ray set and the Cabello 18-ray KS set are mutually reconstructible inside the 24-ray Peres–Mermin eigensystem [6–10]; nevertheless, their context hypergraphs have different two-valued-state spaces.

Section 2 relates the three-dimensional construction for a qutrit (a three-level quantum system) to the Yu–Oh, Harding–Salinas Schmeis, and Cabello diagrams and gives the complete ray and context inventories. Section 4 separates the higher-dimensional forcing constraints from their coordinate completions and then compares the D=4 gadget with the Cabello set. Appendix A records the qutrit MUB convention used throughout.

## The Yu–Oh–Harding–Salinas Schmeis–Cabello Gadget

The 25-ray, 16-context full-context completion of the original 13-ray Yu–Oh (YO) set supports a state-independent noncontextuality inequality for a qutrit [11–13], but it is not itself a KS configuration under Definition 2 because it has 24 separating two-valued states. Cabello’s “triple YO diagram” [14] combines three copies of this 25-ray full-context completion whose center rays form the Fourier basis

$$ \mathcal {B}_1=\{(1,1,1),(1,\omega ,\omega ^2),(1,\omega ^2,\omega )\}, \qquad \omega =\textrm{e}^{2\pi \textrm{i} /3}. $$

**Fig. 1:**
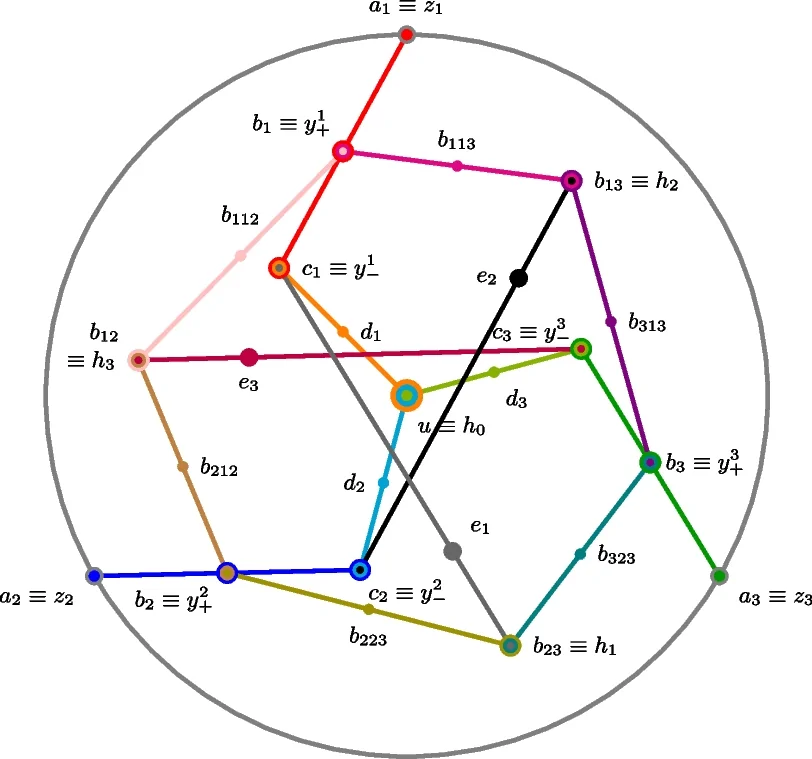
The 25-ray full-context completion of the original 13-ray Yu–Oh set [12], drawn in the Harding and Salinas Schmeis 3-uniform hypergraph scheme [15]

**Fig. 2:**
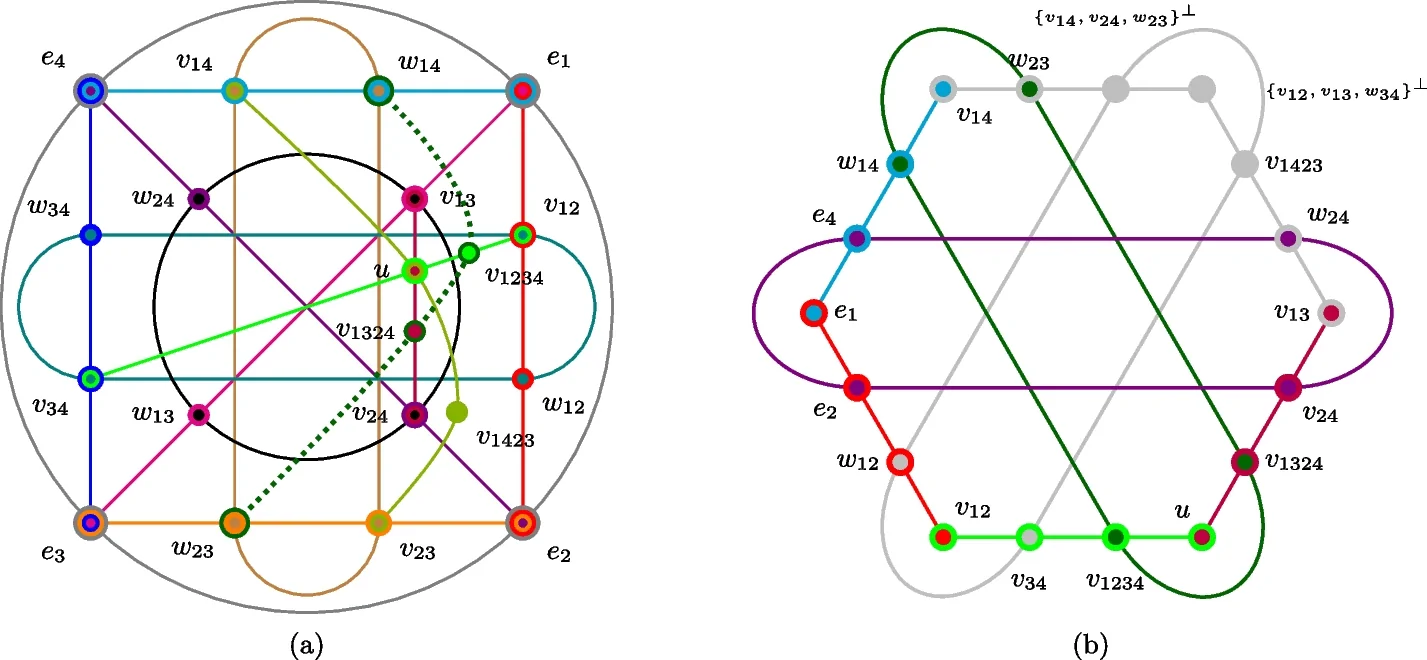
(**a**) Four-dimensional extension of the Harding–Salinas Schmeis hypergraph notation [15]. Solid curves show imposed contexts. The dotted dark-green curve is the equality-induced context $\mathcal {E}_3=\{w_{14},w_{23},v_{1234},v_{1324}\}$; the other contexts of the complete 20-ray orthogonality hypergraph are listed in the text. (**b**) The Cabello 18-ray, 9-context KS configuration [10], with labels from Ref. [2]. Shared rays are relabeled by the gadget notation; overall nonzero scalar factors are immaterial. The notation $\{v_i,v_j,v_k\}^{\perp }$ denotes the unique ray orthogonal to the span of three independent rays. The drawings record the stated incidence relations; no lattice-theoretic conclusion is inferred from the planar layout alone

Relative to the computational rim $\mathcal {B}_0=\{(1,0,0),(0,1,0),(0,0,1)\}$, this is one member of a complete set of four MUBs. Replacing $\mathcal {B}_1$ by either of the other two noncomputational MUBs listed in Appendix A gives an isomorphic 69-ray, 50-context KS configuration. The same incidence pattern appears in the Harding–Salinas Schmeis construction [15, Fig. 2 and Theorem 2.6]; Fig. 1 uses their Greechie-like notation to display the common structure.

### Guide to the Construction

The calculation has four stages. First, index the nine seed vectors by j=1,…,9, and denote the seed vector with index *j* by $\sigma _j$, where

$$ \begin{aligned}&(\sigma _1,\sigma _2,\sigma _3)=((1,1,1),(1,\omega ,\omega ^2),(1,\omega ^2,\omega )),\\&(\sigma _4,\sigma _5,\sigma _6)=((1,\omega ,\omega ),(1,\omega ^2,1),(1,1,\omega ^2)),\\&(\sigma _7,\sigma _8,\sigma _9)=((1,\omega ^2,\omega ^2),(1,\omega ,1),(1,1,\omega )). \end{aligned} $$

The three displayed triples are, respectively, the bases $\mathcal {B}_1,\mathcal {B}_2,\mathcal {B}_3$. Second, the 25 maps in Table 1 are applied to every seed, producing 225 labelled vectors. Third, scalar multiples are identified projectively, leaving the 165 rays in Table 2; all mutually orthogonal triples among those rays are then enumerated, giving the 130 contexts in Table 3. Fourth, every ray and context is classified by the complete set of seed lineages from which it can be generated.

This last step is essential. The three 69-ray lineages have a 21-ray intersection, and no ray belongs to exactly two lineages. Each lineage therefore consists of the same 21 common rays plus 48 exclusive rays. At the context level, 10 contexts are common to all three lineages and each lineage contributes 40 additional contexts, so 10+3·40=130. The provenance structure is therefore symmetric.

**Table 1:** *Systematic generation of the vector set for contextuality analysis.* This table details the complete set of 225 vectors that form the basis of our structural analysis. A family of 25 algebraic generation rules *F* (rows) is applied to nine seed vectors $\sigma _j$, indexed by j=1,…,9. Column *j* contains the vectors generated from the seed $\sigma _j$. Columns 1–3, 4–6, and 7–9 correspond, respectively, to the three noncomputational MUBs $\mathcal {B}_1=\{\sigma _1,\sigma _2,\sigma _3\}$, $\mathcal {B}_2=\{\sigma _4,\sigma _5,\sigma _6\}$, $\mathcal {B}_3=\{\sigma _7,\sigma _8,\sigma _9\}$. Each basis generates a 69-ray, 50-context KS configuration. The entry in column *j* is $F(\sigma _j)$ and receives the inventory label $F_j$; for example, $d_1(\sigma _4)$ is labelled $d_{14}$. The identity rule $u\equiv h_0$ gives $u_j=\sigma _j$. The final column counts the number of unique rays produced by each function, highlighting the differing projective variety of the constructions. The 165 unique rays drawn from this set form the inventory used to identify orthogonal bases, hyperedges, or contexts. The row symbols follow the Harding–Salinas Schmeis scheme [15], with equivalences to the Yu–Oh numbering scheme [12]

Seed	$\mathcal {B}_1$			$\mathcal {B}_2$			$\mathcal {B}_3$			
rule	j=1	j=2	j=3	j=4	j=5	j=6	j=7	j=8	j=9	#
$a_1\equiv z_1$	(1, 0, 0)	(1, 0, 0)	(1, 0, 0)	(1, 0, 0)	(1, 0, 0)	(1, 0, 0)	(1, 0, 0)	(1, 0, 0)	(1, 0, 0)	1
$a_2\equiv z_2$	(0, 1, 0)	(0, 1, 0)	(0, 1, 0)	(0, 1, 0)	(0, 1, 0)	(0, 1, 0)	(0, 1, 0)	(0, 1, 0)	(0, 1, 0)	1
$a_3\equiv z_3$	(0, 0, 1)	(0, 0, 1)	(0, 0, 1)	(0, 0, 1)	(0, 0, 1)	(0, 0, 1)	(0, 0, 1)	(0, 0, 1)	(0, 0, 1)	1
$u\equiv h_0$	(1, 1, 1)	$(1,\omega ,\omega ^2)$	$(1,\omega ^2,\omega )$	$(1,\omega ,\omega )$	$(1,\omega ^2,1)$	$(1,1,\omega ^2)$	$(1,\omega ^2,\omega ^2)$	(1,ω,1)	(1,1,ω)	9
$b_1\equiv y_+^1$	(0, 1, 1)	$(0,\omega ,\omega ^2)$	$(0,\omega ^2,\omega )$	$(0,\omega ,\omega )$	$(0,\omega ^2,1)$	$(0,1,\omega ^2)$	$(0,\omega ^2,\omega ^2)$	(0,ω,1)	(0,1,ω)	3
$b_2\equiv y_+^2$	(1, 0, 1)	$(1,0,\omega ^2)$	(1,0,ω)	(1,0,ω)	(1, 0, 1)	$(1,0,\omega ^2)$	$(1,0,\omega ^2)$	(1, 0, 1)	(1,0,ω)	3
$b_3\equiv y_+^3$	(1, 1, 0)	(1,ω,0)	$(1,\omega ^2,0)$	(1,ω,0)	$(1,\omega ^2,0)$	(1, 1, 0)	$(1,\omega ^2,0)$	(1,ω,0)	(1, 1, 0)	3
$c_1\equiv y_-^1$	(0,1,-1)	$(0,\omega ,-\omega ^2)$	$(0,\omega ^2,-\omega )$	$(0,\omega ^2,-\omega ^2)$	(0,1,-ω)	(0,ω,-1)	$(0,\omega ,-\omega )$	$(0,1,-\omega ^2)$	$(0,\omega ^2,-1)$	3
$c_2\equiv y_-^2$	(-1,0,1)	(-ω,0,1)	$(-\omega ^2,0,1)$	$(-\omega ^2,0,1)$	(-1,0,1)	(-ω,0,1)	(-ω,0,1)	(-1,0,1)	$(-\omega ^2,0,1)$	3
$c_3\equiv y_-^3$	(1,-1,0)	$(\omega ^2,-1,0)$	(ω,-1,0)	$(\omega ^2,-1,0)$	(ω,-1,0)	(1,-1,0)	(ω,-1,0)	$(\omega ^2,-1,0)$	(1,-1,0)	3
$d_1$	(-2,1,1)	$(-2,\omega ,\omega ^2)$	$(-2,\omega ^2,\omega )$	$(-2,\omega ,\omega )$	$(-2,\omega ^2,1)$	$(-2,1,\omega ^2)$	$(-2,\omega ^2,\omega ^2)$	(-2,ω,1)	(-2,1,ω)	9
$d_2$	(1,-2,1)	$(\omega ^2,-2,\omega )$	$(\omega ,-2,\omega ^2)$	$(\omega ^2,-2,1)$	$(\omega ,-2,\omega )$	$(1,-2,\omega ^2)$	(ω,-2,1)	$(\omega ^2,-2,\omega ^2)$	(1,-2,ω)	9
$d_3$	(1,1,-2)	$(\omega ,\omega ^2,-2)$	$(\omega ^2,\omega ,-2)$	$(\omega ^2,1,-2)$	$(1,\omega ^2,-2)$	$(\omega ,\omega ,-2)$	(ω,1,-2)	(1,ω,-2)	$(\omega ^2,\omega ^2,-2)$	9
$b_{12}\equiv h_3$	(1,1,-1)	$(1,\omega ,-\omega ^2)$	$(1,\omega ^2,-\omega )$	$(\omega ,\omega ^2,-\omega ^2)$	$(\omega ,1,-\omega )$	$(\omega ,\omega ,-1)$	$(\omega ^2,\omega ,-\omega )$	$(\omega ^2,1,-\omega ^2)$	$(\omega ^2,\omega ^2,-1)$	9
$b_{13}\equiv h_2$	(1,-1,1)	$(1,-\omega ,\omega ^2)$	$(1,-\omega ^2,\omega )$	$(\omega ,-\omega ^2,\omega ^2)$	$(\omega ,-1,\omega )$	$(\omega ,-\omega ,1)$	$(\omega ^2,-\omega ,\omega )$	$(\omega ^2,-1,\omega ^2)$	$(\omega ^2,-\omega ^2,1)$	9
$b_{23}\equiv h_1$	(-1,1,1)	$(-1,\omega ,\omega ^2)$	$(-1,\omega ^2,\omega )$	$(-\omega ,\omega ^2,\omega ^2)$	$(-\omega ,1,\omega )$	$(-\omega ,\omega ,1)$	$(-\omega ^2,\omega ,\omega )$	$(-\omega ^2,1,\omega ^2)$	$(-\omega ^2,\omega ^2,1)$	9
$e_1$	(1, 2, 1)	$(\omega ^2,2,\omega )$	$(\omega ,2,\omega ^2)$	$(\omega ,2\omega ^2,\omega ^2)$	$(1,2\omega ^2,1)$	$(\omega ^2,2\omega ^2,\omega )$	$(\omega ^2,2\omega ,\omega )$	(1,2ω,1)	$(\omega ,2\omega ,\omega ^2)$	9
$e_2$	(1, 1, 2)	$(\omega ,\omega ^2,2)$	$(\omega ^2,\omega ,2)$	$(\omega ,\omega ^2,2\omega ^2)$	$(\omega ^2,\omega ,2\omega ^2)$	$(1,1,2\omega ^2)$	$(\omega ^2,\omega ,2\omega )$	$(\omega ,\omega ^2,2\omega )$	(1,1,2ω)	9
$e_3$	(2, 1, 1)	$(2,\omega ,\omega ^2)$	$(2,\omega ^2,\omega )$	$(2\omega ^2,1,1)$	$(2\omega ^2,\omega ,\omega ^2)$	$(2\omega ^2,\omega ^2,\omega )$	(2ω,1,1)	$(2\omega ,\omega ^2,\omega )$	$(2\omega ,\omega ,\omega ^2)$	9
$b_{112}$	(2,-1,1)	$(2,-\omega ,\omega ^2)$	$(2,-\omega ^2,\omega )$	$(2,-\omega ,\omega )$	$(2,-\omega ^2,1)$	$(2,-1,\omega ^2)$	$(2,-\omega ^2,\omega ^2)$	(2,-ω,1)	(2,-1,ω)	9
$b_{212}$	(-1,2,1)	$(-1,2\omega ,\omega ^2)$	$(-1,2\omega ^2,\omega )$	$(-1,2\omega ,\omega )$	$(-1,2\omega ^2,1)$	$(-1,2,\omega ^2)$	$(-1,2\omega ^2,\omega ^2)$	(-1,2ω,1)	(-1,2,ω)	9
$b_{113}$	(2,1,-1)	$(2,\omega ,-\omega ^2)$	$(2,\omega ^2,-\omega )$	$(2,\omega ,-\omega )$	$(2,\omega ^2,-1)$	$(2,1,-\omega ^2)$	$(2,\omega ^2,-\omega ^2)$	(2,ω,-1)	(2,1,-ω)	9
$b_{313}$	(-1,1,2)	$(-1,\omega ,2\omega ^2)$	$(-1,\omega ^2,2\omega )$	$(-1,\omega ,2\omega )$	$(-1,\omega ^2,2)$	$(-1,1,2\omega ^2)$	$(-1,\omega ^2,2\omega ^2)$	(-1,ω,2)	(-1,1,2ω)	9
$b_{223}$	(1,2,-1)	$(1,2\omega ,-\omega ^2)$	$(1,2\omega ^2,-\omega )$	$(1,2\omega ,-\omega )$	$(1,2\omega ^2,-1)$	$(1,2,-\omega ^2)$	$(1,2\omega ^2,-\omega ^2)$	(1,2ω,-1)	(1,2,-ω)	9
$b_{323}$	(1,-1,2)	$(1,-\omega ,2\omega ^2)$	$(1,-\omega ^2,2\omega )$	$(1,-\omega ,2\omega )$	$(1,-\omega ^2,2)$	$(1,-1,2\omega ^2)$	$(1,-\omega ^2,2\omega ^2)$	(1,-ω,2)	(1,-1,2ω)	9

**Table 2:** The complete set of 165 projectively distinct rays, with six entries per row. The rightmost index records the seed column in Table 1. The 21 rays common to all three 69-ray lineages are black. The 48 rays exclusive to $\mathcal {B}_1$, $\mathcal {B}_2$, and $\mathcal {B}_3$ are shown in red, green, and blue, respectively

Tab2

**Table 3:** The complete list of 130 orthogonal bases supported by the 165 rays. The invariant provenance counts are stated in Proposition 1

{$a_{11}$, $a_{21}$, $a_{31}$}	{$a_{11}$, $b_{11}$, $c_{11}$}	{$a_{11}$, $b_{12}$, $c_{12}$}	{$a_{11}$, $b_{13}$, $c_{13}$}	{$a_{21}$, $b_{21}$, $c_{21}$}	{$a_{21}$, $b_{22}$, $c_{22}$}
{$a_{21}$, $b_{23}$, $c_{23}$}	{$a_{31}$, $b_{31}$, $c_{31}$}	{$a_{31}$, $b_{32}$, $c_{32}$}	{$a_{31}$, $b_{33}$, $c_{33}$}	{$u_{1}$, $u_{2}$, $u_{3}$}	{$u_{1}$, $c_{11}$, $d_{11}$}
{$u_{1}$, $c_{21}$, $d_{21}$}	{$u_{1}$, $c_{31}$, $d_{31}$}	{$u_{2}$, $c_{12}$, $d_{12}$}	{$u_{2}$, $c_{22}$, $d_{22}$}	{$u_{2}$, $c_{32}$, $d_{32}$}	{$u_{3}$, $c_{13}$, $d_{13}$}
{$u_{3}$, $c_{23}$, $d_{23}$}	{$u_{3}$, $c_{33}$, $d_{33}$}	{$u_{4}$, $u_{5}$, $u_{6}$}	{$u_{4}$, $c_{11}$, $d_{14}$}	{$u_{4}$, $c_{23}$, $d_{24}$}	{$u_{4}$, $c_{32}$, $d_{34}$}
{$u_{5}$, $c_{12}$, $d_{15}$}	{$u_{5}$, $c_{21}$, $d_{25}$}	{$u_{5}$, $c_{33}$, $d_{35}$}	{$u_{6}$, $c_{13}$, $d_{16}$}	{$u_{6}$, $c_{22}$, $d_{26}$}	{$u_{6}$, $c_{31}$, $d_{36}$}
{$u_{7}$, $u_{8}$, $u_{9}$}	{$u_{7}$, $c_{11}$, $d_{17}$}	{$u_{7}$, $c_{22}$, $d_{27}$}	{$u_{7}$, $c_{33}$, $d_{37}$}	{$u_{8}$, $c_{13}$, $d_{18}$}	{$u_{8}$, $c_{21}$, $d_{28}$}
{$u_{8}$, $c_{32}$, $d_{38}$}	{$u_{9}$, $c_{12}$, $d_{19}$}	{$u_{9}$, $c_{23}$, $d_{29}$}	{$u_{9}$, $c_{31}$, $d_{39}$}	{$b_{11}$, $b_{121}$, $b_{1121}$}	{$b_{11}$, $b_{124}$, $b_{1124}$}
{$b_{11}$, $b_{127}$, $b_{1127}$}	{$b_{11}$, $b_{131}$, $b_{1131}$}	{$b_{11}$, $b_{134}$, $b_{1134}$}	{$b_{11}$, $b_{137}$, $b_{1137}$}	{$b_{12}$, $b_{122}$, $b_{1122}$}	{$b_{12}$, $b_{125}$, $b_{1125}$}
{$b_{12}$, $b_{129}$, $b_{1129}$}	{$b_{12}$, $b_{132}$, $b_{1132}$}	{$b_{12}$, $b_{135}$, $b_{1135}$}	{$b_{12}$, $b_{139}$, $b_{1139}$}	{$b_{13}$, $b_{123}$, $b_{1123}$}	{$b_{13}$, $b_{126}$, $b_{1126}$}
{$b_{13}$, $b_{128}$, $b_{1128}$}	{$b_{13}$, $b_{133}$, $b_{1133}$}	{$b_{13}$, $b_{136}$, $b_{1136}$}	{$b_{13}$, $b_{138}$, $b_{1138}$}	{$b_{21}$, $b_{121}$, $b_{2121}$}	{$b_{21}$, $b_{125}$, $b_{2125}$}
{$b_{21}$, $b_{128}$, $b_{2128}$}	{$b_{21}$, $b_{231}$, $b_{2231}$}	{$b_{21}$, $b_{235}$, $b_{2235}$}	{$b_{21}$, $b_{238}$, $b_{2238}$}	{$b_{22}$, $b_{122}$, $b_{2122}$}	{$b_{22}$, $b_{126}$, $b_{2126}$}
{$b_{22}$, $b_{127}$, $b_{2127}$}	{$b_{22}$, $b_{232}$, $b_{2232}$}	{$b_{22}$, $b_{236}$, $b_{2236}$}	{$b_{22}$, $b_{237}$, $b_{2237}$}	{$b_{23}$, $b_{123}$, $b_{2123}$}	{$b_{23}$, $b_{124}$, $b_{2124}$}
{$b_{23}$, $b_{129}$, $b_{2129}$}	{$b_{23}$, $b_{233}$, $b_{2233}$}	{$b_{23}$, $b_{234}$, $b_{2234}$}	{$b_{23}$, $b_{239}$, $b_{2239}$}	{$b_{31}$, $b_{131}$, $b_{3131}$}	{$b_{31}$, $b_{136}$, $b_{3136}$}
{$b_{31}$, $b_{139}$, $b_{3139}$}	{$b_{31}$, $b_{231}$, $b_{3231}$}	{$b_{31}$, $b_{236}$, $b_{3236}$}	{$b_{31}$, $b_{239}$, $b_{3239}$}	{$b_{32}$, $b_{132}$, $b_{3132}$}	{$b_{32}$, $b_{134}$, $b_{3134}$}
{$b_{32}$, $b_{138}$, $b_{3138}$}	{$b_{32}$, $b_{232}$, $b_{3232}$}	{$b_{32}$, $b_{234}$, $b_{3234}$}	{$b_{32}$, $b_{238}$, $b_{3238}$}	{$b_{33}$, $b_{133}$, $b_{3133}$}	{$b_{33}$, $b_{135}$, $b_{3135}$}
{$b_{33}$, $b_{137}$, $b_{3137}$}	{$b_{33}$, $b_{233}$, $b_{3233}$}	{$b_{33}$, $b_{235}$, $b_{3235}$}	{$b_{33}$, $b_{237}$, $b_{3237}$}	{$c_{11}$, $b_{231}$, $e_{31}$}	{$c_{11}$, $b_{234}$, $e_{34}$}
{$c_{11}$, $b_{237}$, $e_{37}$}	{$c_{12}$, $b_{232}$, $e_{32}$}	{$c_{12}$, $b_{235}$, $e_{35}$}	{$c_{12}$, $b_{239}$, $e_{39}$}	{$c_{13}$, $b_{233}$, $e_{33}$}	{$c_{13}$, $b_{236}$, $e_{36}$}
{$c_{13}$, $b_{238}$, $e_{38}$}	{$c_{21}$, $b_{131}$, $e_{11}$}	{$c_{21}$, $b_{135}$, $e_{15}$}	{$c_{21}$, $b_{138}$, $e_{18}$}	{$c_{22}$, $b_{132}$, $e_{12}$}	{$c_{22}$, $b_{136}$, $e_{16}$}
{$c_{22}$, $b_{137}$, $e_{17}$}	{$c_{23}$, $b_{133}$, $e_{13}$}	{$c_{23}$, $b_{134}$, $e_{14}$}	{$c_{23}$, $b_{139}$, $e_{19}$}	{$c_{31}$, $b_{121}$, $e_{21}$}	{$c_{31}$, $b_{126}$, $e_{26}$}
{$c_{31}$, $b_{129}$, $e_{29}$}	{$c_{32}$, $b_{122}$, $e_{22}$}	{$c_{32}$, $b_{124}$, $e_{24}$}	{$c_{32}$, $b_{128}$, $e_{28}$}	{$c_{33}$, $b_{123}$, $e_{23}$}	{$c_{33}$, $b_{125}$, $e_{25}$}
{$c_{33}$, $b_{127}$, $e_{27}$}	{$b_{121}$, $b_{122}$, $b_{123}$}	{$b_{124}$, $b_{125}$, $b_{126}$}	{$b_{127}$, $b_{128}$, $b_{129}$}	{$b_{131}$, $b_{132}$, $b_{133}$}	{$b_{134}$, $b_{135}$, $b_{136}$}
{$b_{137}$, $b_{138}$, $b_{139}$}	{$b_{231}$, $b_{232}$, $b_{233}$}	{$b_{234}$, $b_{235}$, $b_{236}$}	{$b_{237}$, $b_{238}$, $b_{239}$}		

We work projectively throughout and use the Harding–Salinas Schmeis labels, augmented where needed by the symbols $e_i$. The full tables make the computational claims reproducible, while the propositions below isolate the conclusions needed later.

## Global Total Number of 165 Non-Collinear Vectors in 130 Orthogonal Bases

A total of 165 unique, non-collinear vectors were identified from the analysis of the nine seed vectors $\sigma _1,\ldots ,\sigma _9$ in Table 1. These vectors, which form the basis for constructing orthogonal triples, are enumerated in Table 2. The coordinate system is based on the complex third root of unity, $\omega = \textrm{e}^{2\pi \textrm{i}{}/3}$.

This vector set is not closed with respect to the complex “cross product” [15] (sometimes referred to as Hermitian cross product) $u\times v = (\overline{u_2v_3-u_3v_2},\overline{u_3v_1-u_1v_3},\overline{u_1v_2-u_2v_1})$ of two vectors $u=(u_1,u_2,u_3)$ and $v=(v_1,v_2,v_3)$ in $\mathbb {R}^3$ or $\mathbb {C}^3$ because, say, $a_{11} \times d_{21} = (1,0,0) \times (1,-2,1) = -(0,1,2)$, which is not collinear with any vector of the set of globally non-collinear vectors.

### Symmetric Provenance Decomposition

For r=1,2,3, let $\mathcal {S}_r$ be the projective ray set generated from the three seeds in $\mathcal {B}_r$, and let $\mathcal {E}_r$ be its set of orthogonal triples.

#### Proposition 1

(Ray and context inventory) The generated families satisfy

$$ |\mathcal {S}_r|=69,\qquad |\mathcal {S}_1\cap \mathcal {S}_2\cap \mathcal {S}_3|=21, \qquad |\mathcal {S}_r\setminus (\mathcal {S}_s\cup \mathcal {S}_t)|=48, $$

where $\{r,s,t\}=\{1,2,3\}$, and no ray belongs to exactly two lineages. Their union has 165 rays. Likewise, each $\mathcal {E}_r$ contains 50 contexts, 10 contexts are common to all three lineages, and each lineage contributes 40 further contexts. The union therefore contains 10+3·40=130 contexts. Exactly four contexts in each lineage consist entirely of lineage-exclusive rays.

#### Proof

Each nonzero generated vector is divided by its first nonzero coordinate to obtain a canonical projective representative. Equality is then decided using $1+\omega +\omega ^2=0$. This gives 69 representatives per lineage and the overlap counts above. Pairwise Hermitian inner products are evaluated exactly in $\mathbb {Q}(\omega )$; triangles in the resulting orthogonality graph are precisely the contexts. The exhaustive search gives 50 contexts per lineage and 130 in the union. Finally, direct enumeration of maps $s:V\rightarrow \{0,1\}$ with one 1 per context gives no two-valued state for any of the three 69-ray, 50-context lineages. □

The symmetric provenance statement depends on the complete sets of generating lineages and is independent of the display labels assigned to the rays.

## Higher-Dimensional Forcing Gadgets

In $\mathcal {P}(\mathbb {C}^3)$, two independent rays determine a unique orthogonal ray. For D≥4, their orthogonal complement has dimension D-2, so additional connector contexts are required to obtain a rigid finite construction.

### Definition 3

(Unbiased vector) A normalized vector *u* is maximally unbiased relative to an orthonormal basis $\mathcal {B}$ if

$$ |\langle e_j,u\rangle |^2=1/D\quad (j=1,\ldots ,D). $$

For $u=(x_1,\ldots ,x_D)$ in the computational basis, this is equivalent to $|x_1|=\cdots =|x_D|$.

The D=4 and D=5 constructions have three logically separate parts: (i) a scaffold of coordinate-supported minors, (ii) connector rays whose orthogonality to the center gives equations in $|x_j|^2$, and (iii) completion vectors that turn the required orthogonal pairs into full contexts. The propositions below state the forcing constraints; coordinate inventories and exact enumeration checks are given afterward.

### Notation and Conventions

Fix an unknown center vector $u = (x_1,\dots ,x_D) \in \mathbb {C}^D$ with $x_j \ne 0$ for all *j*, and use the standard Hermitian inner product

$$ \langle v,w\rangle = \sum _m \overline{v_m}\, w_m. $$

We use vectors to represent the one-dimensional subspaces they span and freely scale them when forming blocks, with the understanding that they must be normalized to form a literal orthonormal basis.

### An Explicit Gadget for D=4

First, take the computational (Cartesian) basis

$$\begin{aligned} e_1&=(1,0,0,0),&e_2 =(0,1,0,0),\\ e_3&=(0,0,1,0),&e_4 =(0,0,0,1), \end{aligned}$$

and place the context $\mathcal {B}_{\textrm{comp}}=\{e_1,e_2,e_3,e_4\}$ on the rim of Fig. 2(a).

In the second construction stage define, for each unordered pair $\{i,j\}\subset \{1,2,3,4\}$, pair minors from a Levi-Civita contraction:

$$\begin{aligned} v_{12}&= (0,0,\overline{x_4},-\overline{x_3}),&v_{13} = (0,-\overline{x_4},0,\overline{x_2}), \\ v_{14}&= (0,\overline{x_3},-\overline{x_2},0),&v_{23} = (\overline{x_4},0,0,-\overline{x_1}), \\ v_{24}&= (-\overline{x_3},0,\overline{x_1},0),&v_{34} = (\overline{x_2},-\overline{x_1},0,0). \end{aligned}$$

Each $v_{ij}$ vanishes in coordinates *i*, *j* and satisfies $\langle v_{ij},u\rangle =0$ by direct cancellation.

In the third step, choose a vector $w_{ij}$ with the same support as $v_{ij}$ and orthogonal to it, that is, $\langle v_{ij},w_{ij}\rangle =0$:

$$\begin{aligned} w_{12}&=(0,0,x_3,x_4),&w_{13} =(0,x_2,0,x_4), \\ w_{14}&=(0,x_2,x_3,0),&w_{23} =(x_1,0,0,x_4), \\ w_{24}&=(x_1,0,x_3,0),&w_{34} =(x_1,x_2,0,0). \end{aligned}$$

For each of the $$\left( {\begin{array}{c}4\\ 2\end{array}}\right) =6$$ pairs $\{i,j\}$, the set

$$ \mathcal {B}_{ij}=\{e_i, e_j, v_{ij}, w_{ij}\} $$

is an orthogonal basis (block, context).

In a fourth step, define three connector vectors

$$\begin{aligned} v_{1234}&=(x_1,x_2,-x_3,-x_4),\\ v_{1324}&=(x_1,-x_2,x_3,-x_4),\\ v_{1423}&=(x_1,-x_2,-x_3,x_4). \end{aligned}$$

Whenever *u* is orthogonal to one of these connector vectors, the two-dimensional complement of their span supplies completion rays $h_1,h_2$. Thus the three connector contexts may be written

$$\begin{aligned} \mathcal {C}_{(a)}&=\{u,v_{1234},h^{(a)}_1,h^{(a)}_2\},&\mathcal {C}_{(b)}&=\{u,v_{1324},h^{(b)}_1,h^{(b)}_2\},\\ \mathcal {C}_{(c)}&=\{u,v_{1423},h^{(c)}_1,h^{(c)}_2\}. \end{aligned}$$

#### Proposition 2

(D=4 forcing constraint) Membership of *u* and the three connector rays in the contexts above forces *u* to be maximally unbiased relative to $\mathcal {B}_{\textrm{comp}}$.

#### Proof

The three required inner products vanish exactly when

1 $$\begin{aligned} |x_1|^2+|x_2|^2&=|x_3|^2+|x_4|^2, \end{aligned}$$

2 $$\begin{aligned} |x_1|^2+|x_3|^2&=|x_2|^2+|x_4|^2, \end{aligned}$$

3 $$\begin{aligned} |x_1|^2+|x_4|^2&=|x_2|^2+|x_3|^2. \end{aligned}$$

Subtracting pairs of equations gives $|x_2|^2=|x_3|^2=|x_4|^2$; substitution gives $|x_1|^2=|x_2|^2$. Since the moduli are nonnegative, $|x_1|=|x_2|=|x_3|=|x_4|$. After normalization, every squared modulus is 1/4. □

#### Automatic and Equality-Induced Contexts

Orthogonalities due only to disjoint support, such as $v_{12}\perp v_{34}$, do not constrain *u*. For the concrete 20-ray representation with u=(1,1,1,1), the computational context, six pair contexts, and three connector contexts give 10 imposed contexts. An exhaustive orthogonality search finds exactly seven further contexts. Three follow from disjoint support:

$$\begin{aligned} \mathcal {B}_{1234}&=\{v_{12},w_{12},v_{34},w_{34}\},\\ \mathcal {B}_{1324}&=\{v_{13},w_{13},v_{24},w_{24}\},\\ \mathcal {B}_{1423}&=\{v_{14},w_{14},v_{23},w_{23}\}. \end{aligned}$$

The remaining four use the equal-modulus relations from Proposition 2:

$$\begin{aligned} \mathcal {C}_0&=\{u,v_{1234},v_{1324},v_{1423}\},\\ \mathcal {E}_1&=\{w_{12},w_{34},v_{1324},v_{1423}\},\\ \mathcal {E}_2&=\{w_{13},w_{24},v_{1234},v_{1423}\},\\ \mathcal {E}_3&=\{w_{14},w_{23},v_{1234},v_{1324}\}. \end{aligned}$$

Hence the complete orthogonality hypergraph of the concrete 20-ray set has 17 contexts, not 13. Exact enumeration gives 16 two-valued states, and these states separate every pair of rays; the complete 20-ray hypergraph is therefore not a KS configuration.

**Table 4:** Comparison of the 20-ray gadget and the Cabello 18-ray set [2, 10]. Both lie in the 24-ray Peres–Mermin eigensystem [6–9, 16]. Fifteen rays are shared projectively. Each of the five gadget-only rays and each of the three Cabello-only rays is the unique orthogonal complement of a three-dimensional span generated by the other set, establishing mutual reconstructibility

Gadget label	Gadget vector	Cabello diagram label	Vector construction / Definition
*u*	(1, 1, 1, 1)	$v_{56}$	(1, 1, 1, 1)
$e_1$	(1, 0, 0, 0)	$v_{12}$	(1, 0, 0, 0)
$e_2$	(0, 1, 0, 0)	$v_{18}$	(0, 1, 0, 0)
$e_3$	(0, 0, 1, 0)	(Constructed)	$\{v_{12}, v_{18}, v_{28}\}^\perp$
$e_4$	(0, 0, 0, 1)	$v_{28}$	(0, 0, 0, 1)
$v_{12}$	(0,0,1,-1)	$v_{16}$	(0,0,1,-1)
$v_{13}$	(0,-1,0,1)	$v_{45}$	(0,1,0,-1)
$v_{14}$	(0,1,-1,0)	$v_{23}$	(0,1,-1,0)
$v_{23}$	(1,0,0,-1)	(Constructed)	$\{v_{18},v_{23},v_{39}\}^\perp$
$v_{24}$	(-1,0,1,0)	$v_{58}$	(1,0,-1,0)
$v_{34}$	(1,-1,0,0)	$v_{67}$	(1,-1,0,0)
$w_{12}$	(0, 0, 1, 1)	$v_{17}$	(0, 0, 1, 1)
$w_{13}$	(0, 1, 0, 1)	(Constructed)	$\{v_{12},v_{45},v_{58}\}^\perp$
$w_{14}$	(0, 1, 1, 0)	$v_{29}$	(0, 1, 1, 0)
$w_{23}$	(1, 0, 0, 1)	$v_{39}$	(1, 0, 0, 1)
$w_{24}$	(1, 0, 1, 0)	$v_{48}$	(1, 0, 1, 0)
$w_{34}$	(1, 1, 0, 0)	(Constructed)	$\{v_{28},v_{16},v_{67}\}^\perp$
$v_{1234}$	(1,1,-1,-1)	$v_{69}$	(1,1,-1,-1)
$v_{1324}$	(1,-1,1,-1)	$v_{59}$	(1,-1,1,-1)
$v_{1423}$	(1,-1,-1,1)	(Constructed)	$\{v_{23},v_{17},v_{48}\}^\perp$
(Constructed)	$\{v_{12}, v_{13}, w_{34}\}^\perp$	$v_{34}$	(-1,1,1,1)
(Constructed)	$\{v_{14}, v_{24}, w_{23}\}^\perp$	$v_{37}$	(1,1,1,-1)
(Constructed)	$\{v_{13},v_{23},w_{12}\}^\perp$	$v_{47}$	(1,1,-1,1)

#### Concrete D=4 Example: u=(1,1,1,1)

Take the center vector u=(1,1,1,1) and the rim vectors

$$\begin{aligned} e_1&=(1,0,0,0),&e_2 =(0,1,0,0),\\ e_3&=(0,0,1,0),&e_4 =(0,0,0,1). \end{aligned}$$

The pair minors and their complementary vectors become:

$$\begin{aligned} v_{12}&=(0,0,1,-1),&w_{12}&=(0,0,1,1),\\ v_{13}&=(0,-1,0,1),&w_{13}&=(0,1,0,1),\\ v_{14}&=(0,1,-1,0),&w_{14}&=(0,1,1,0),\\ v_{23}&=(1,0,0,-1),&w_{23}&=(1,0,0,1),\\ v_{24}&=(-1,0,1,0),&w_{24}&=(1,0,1,0),\\ v_{34}&=(1,-1,0,0),&w_{34}&=(1,1,0,0). \end{aligned}$$

Each pair block

$$ \mathcal {B}_{ij}=\{e_i, e_j, v_{ij}, w_{ij}\} $$

forms an ONB after normalizing $v_{ij}$ and $w_{ij}$.

The connector vectors are

$$\begin{aligned} v_{1234}&=(1,1,-1,-1),\\ v_{1324}&=(1,-1,1,-1),\\ v_{1423}&=(1,-1,-1,1), \end{aligned}$$

and they are all orthogonal to u=(1,1,1,1) by inspection.

A convenient explicit completion of the connector blocks can be formed using some of the existing minor vectors:

$$\begin{aligned} C_{(a)}&=\{u, v_{1234}, v_{34}, v_{12}\},\\ C_{(b)}&=\{u, v_{1324}, v_{24}, v_{13}\},\\ C_{(c)}&=\{u, v_{1423}, v_{23}, v_{14}\}. \end{aligned}$$

Each of these sets is composed of four mutually orthogonal vectors. For example, in $C_{(a)}$, the vectors $v_{34}$ and $v_{12}$ have disjoint support, making them orthogonal. The inner products $\langle v_{1234}, v_{34} \rangle$ and $\langle v_{1234}, v_{12} \rangle$ are zero, as are all other inner products involving *u*. To create literal ONBs, the vectors should be normalized.

Together with $\mathcal {B}_{\textrm{comp}}$, the six pair contexts and three connector contexts form the 10-context scaffold that enforces Proposition 2. The equal-modulus condition also creates $\mathcal {C}_0$ and the three contexts $\mathcal {E}_1,\mathcal {E}_2,\mathcal {E}_3$ listed above. For example, $\langle w_{14},v_{1234}\rangle =|x_2|^2-|x_3|^2=0$; $\mathcal {E}_3$ is the dotted dark-green curve in Fig. 2(a).

#### Mutual Reconstruction Inside the Peres–Mermin Eigensystem

Both ray sets are subsets of the 24-ray Peres–Mermin eigensystem [9, 16]. They share 15 rays projectively. Table 4 gives rank-three orthogonal-complement constructions for the five gadget-only rays and the three Cabello-only rays. Hence each ray set generates the other under the operation “take the unique ray orthogonal to three independent rays.” This is the precise sense in which we call them informationally equivalent.

Informational equivalence does not imply equality of context hypergraphs. The complete 20-ray orthogonality hypergraph has 17 contexts and 16 separating two-valued states. The Cabello 18-ray hypergraph selects nine contexts and has no two-valued state; its parity argument makes it a KS configuration [10]. The contrast is therefore not a consequence of “more contexts allowing loopholes”—adding constraints cannot create two-valued states—but of the two constructions selecting different rays and different contexts from their common 24-ray parent.

#### Context Completion and Two-Valued States

The context choice within the 20-ray set can itself be tracked explicitly. Let

$$ \mathfrak {B}_{10}=\{\mathcal {B}_{\textrm{comp}},\mathcal {B}_{ij}\ (1\le i<j\le 4), \mathcal {C}_{(a)},\mathcal {C}_{(b)},\mathcal {C}_{(c)}\} $$

be the 10 imposed contexts, and let

$$ \mathfrak {B}_{13}=\mathfrak {B}_{10}\cup \{\mathcal {B}_{1234},\mathcal {B}_{1324},\mathcal {B}_{1423}\}. $$

Exact enumeration shows that both hypergraphs have the same 36 two-valued states, and those states are separating. The three disjoint-support contexts therefore record exclusions already obeyed by every state of $\mathfrak {B}_{10}$; they do not remove an assignment. Adding the four equality-induced contexts gives the complete set

$$ \mathfrak {B}_{17}=\mathfrak {B}_{13}\cup \{\mathcal {C}_0,\mathcal {E}_1,\mathcal {E}_2,\mathcal {E}_3\}, $$

which has 16 separating two-valued states. This nested comparison makes the monotonicity transparent: adding contexts can preserve or reduce the state set, but cannot enlarge it. It also avoids attributing KS contextuality to an incomplete incidence structure merely because some available contexts were omitted.

The apparent asymmetry of Fig. 2(a) is mainly graphical and reflects the selected 20-ray subset. Both it and the Cabello 18-ray set sit inside the more symmetric 24-ray, 24-context Peres configuration [17, 18]; neither subset needs to inherit all symmetries of the parent.

### A D=5 Scaffold and Forcing Constraint

Let $\varepsilon _{abcde}$ be a totally antisymmetric tensor with $\varepsilon _{12345}=1$. For each triple $\{i,j,k\}$ define

$$ (v_{ijk})_m=\sum _{\ell =1}^5\varepsilon _{mijk\ell }\,\overline{x_\ell }. $$

The vector $v_{ijk}$ vanishes in coordinates *i*, *j*, *k* and satisfies $\langle v_{ijk},u\rangle =0$ by antisymmetry. Choose $w_{ijk}$ on the complementary two coordinates with $v_{ijk}\perp w_{ijk}$; then

$$ \mathcal {B}_{ijk}=\{e_i,e_j,e_k,v_{ijk},w_{ijk}\} $$

is a scaffold context.

The four connector rays are represented by

$$\begin{aligned} g_1&=(x_1,0,0,0,-x_5),&g_2&=(0,x_2,0,0,-x_5),\\ g_3&=(0,0,x_3,0,-x_5),&g_4&=(0,0,0,x_4,-x_5). \end{aligned}$$

Whenever $u\perp g_i$, three rays spanning ${\text {span}}\{u,g_i\}^{\perp }$ complete the connector context $\mathcal {C}_i=\{u,g_i,h^{(i)}_1,h^{(i)}_2,h^{(i)}_3\}$.

#### Proposition 3

(D=5 forcing constraint) The four connector contexts force a normalized center vector *u* to be maximally unbiased relative to the computational basis.

#### Proof

For i=1,2,3,4,

$$ \langle u,g_i\rangle =|x_i|^2-|x_5|^2. $$

Thus the four orthogonality conditions imply $|x_1|=\cdots =|x_5|$; normalization makes every squared modulus 1/5. □

## Summary and Conclusion

The qutrit construction yields 165 projectively distinct rays and exactly 130 contexts. Correct provenance accounting is symmetric: the three 69-ray, 50-context KS lineages share 21 rays and 10 contexts, and each adds 48 exclusive rays and 40 lineage-specific contexts. This places the triple of 25-ray full-context Yu–Oh completions, Harding–Salinas Schmeis, and Cabello descriptions in a common MUB-based framework without assigning shared rays to an arbitrary first lineage.

In D=4 and D=5, the forcing mechanism is explicit at the level of constraints: connector orthogonalities yield linear equations in the squared coordinate moduli and hence maximal unbiasedness. For the concrete D=4 realization, the complete 20-ray orthogonality hypergraph has 17 contexts and 16 separating two-valued states. Its ray set is mutually reconstructible with the Cabello 18-ray set inside the 24-ray Peres–Mermin eigensystem, but the Cabello nine-context hypergraph is uncolorable. Ray-set closure and context incidence are therefore distinct pieces of data.

The main methodological conclusion is that contextuality claims should specify the incidence hypergraph being tested and, when completeness is intended, enumerate every context supported by the chosen rays. Restricting attention to intertwining rays or to a selected subset of available contexts may define a legitimate reduced scenario, but its two-valued-state properties should not be transferred to a different completion without a separate check.

## Data Availability

No datasets were generated or analysed during the current study.

## Appendix A: Mutually Unbiased Bases in $\mathbb {C}^3$

For introductions to MUBs, see Refs. [19–24]. Write

$$ \omega =\textrm{e}^{2\pi \textrm{i} /3},\qquad \omega ^3=1,\qquad 1+\omega +\omega ^2=0, \qquad \overline{\omega }=\omega ^2. $$

In D=3, a complete MUB family contains four bases [25]. Omitting the common normalization factor $1/\sqrt{3}$ from the last three bases, we use

A1 $$\begin{aligned} \mathcal {B}_0&=\{(1,0,0),(0,1,0),(0,0,1)\}, \end{aligned}$$

A2 $$\begin{aligned} \mathcal {B}_1&=\{(1,1,1),(1,\omega ,\omega ^2),(1,\omega ^2,\omega )\}, \end{aligned}$$

A3 $$\begin{aligned} \mathcal {B}_2&=\{(1,\omega ,\omega ),(1,\omega ^2,1),(1,1,\omega ^2)\}, \end{aligned}$$

A4 $$\begin{aligned} \mathcal {B}_3&=\{(1,\omega ^2,\omega ^2),(1,\omega ,1),(1,1,\omega )\}. \end{aligned}$$

Here $\mathcal {B}_0$ is computational and $\mathcal {B}_1$ is the Fourier basis. Direct use of $1+\omega +\omega ^2=0$ verifies orthogonality within each basis and $|\langle b,b'\rangle |^2=1/3$ between normalized vectors from different bases. The ordering of $\mathcal {B}_{1} ,\mathcal {B}_2$ and $\mathcal {B}_3$ agrees with the seed-vector groups assigned to columns 1–3, 4–6, and 7–9 of Table 1.

For the higher-dimensional argument, no catalogue of a complete MUB family is required: Propositions 2 and 3 use only the criterion $|x_1|=\cdots =|x_D|$ for one vector to be maximally unbiased relative to the computational basis.
